# One-stop diagnostic breast clinics: how often are breast cancers missed?

**DOI:** 10.1038/sj.bjc.6605082

**Published:** 2009-05-19

**Authors:** P Britton, S W Duffy, R Sinnatamby, M G Wallis, S Barter, M Gaskarth, A O'Neill, C Caldas, J D Brenton, P Forouhi, G C Wishart

**Affiliations:** 1 Department of Radiology, Cambridge Breast Unit, Box 97, Cambridge University Hospitals NHS Foundation Trust, Cambridge CB2 2QQ, UK; 2 Cancer Research UK Centre for Epidemiology, Mathematics and Statistics, Wolfson Institute of Preventive Medicine, Barts and the London School of Medicine and Dentistry, London Charterhouse Square, London EC1 M 6BQ, UK; 3 Department of Oncology, University of Cambridge and Oncology Centre, Addenbrooke's Hospital, Cambridge CB2 2QQ, UK; 4 Department of Surgery, Cambridge Breast Unit, Cambridge University Hospitals NHS Foundation Trust, Cambridge CB2 2QQ, UK

**Keywords:** breast cancer, breast diseases, diagnosis, diagnostic errors

## Abstract

The aim of this study was to estimate the number of patients discharged from a symptomatic breast clinic who subsequently develop breast cancer and to determine how many of these cancers had been ‘missed’ at initial assessment. Over a 3-year period, 7004 patients were discharged with a nonmalignant diagnosis. Twenty-nine patients were subsequently diagnosed with breast cancer over the next 36 months. This equates to a symptomatic ‘interval’ cancer rate of 4.1 per 1000 women in the 36 months after initial assessment (0.9 per 1000 women within 12 months, 2.6 per 1000 women within 24 months). The lowest sensitivity of initial assessment was seen in patients of 40–49 years of age, and these patients present the greatest imaging and diagnostic challenge. Following multidisciplinary review, a consensus was reached on whether a cancer had been missed or not. No delay occurred in 10 patients (35%) and probably no delay in 7 patients (24%). Possible delay occurred in three patients (10%) and definite delay in diagnosis (i.e., a ‘missed’ cancer) occurred in only nine patients (31%). The overall diagnostic accuracy of ‘triple’ assessment is 99.6% and the ‘missed’ cancer rate is 1.7 per 1000 women discharged.

The majority of breast cancers in the United Kingdom are detected in symptomatic women referred by their general practitioners to breast clinics for further investigation (NHS Information Centre, 2005; UK Statistics Authority, 2005). Breast cancer diagnostic services have become increasingly streamlined and patients are frequently seen by multidisciplinary teams (MDTs) in dedicated breast clinics (Potter *et al*, 2007). The standard diagnostic process is referred to as ‘triple assessment’, namely expert clinical breast examination, breast imaging (a combination of mammography and ultrasound (US), or both) and, when necessary, needle biopsy (Britton and Sinnatamby, 2007). The primary aim of ‘symptomatic’ clinics is to separate patients with breast cancer from the majority (in the region of 93%) who do not (Barber *et al*, 2004). Inevitably, however, some patients will be diagnosed with breast cancer having been recently discharged from a breast clinic with a normal or benign diagnosis. Although some of these cancers will have developed *de novo*, others will have been ‘missed’ at initial assessment resulting in a delay in diagnosis. Such delay causes patient anxiety, may affect the clinical outcome and is a major cause of medical malpractice in the United Kingdom (Barber *et al*, 2004).

When the National Health Services Breast Screening Programme (NHSBSP) was set up in 1988, its performance was managed by a strong quality assurance programme using nationally set targets and regional quality assurance team inspections (NHS Breast Screening Programme, 2000, 2005a, 2005b). Screened women with a normal result who subsequently develop breast cancer before their next screening invitation are referred to as having developed an interval cancer. A screening programme interval cancer rate can be used as a performance indicator, and NHSBSP standards for interval cancer rates are published (NHS Breast Screening Programme, 2005b).

The ‘symptomatic’ service has far less rigorous monitoring. Following the publication of the updated National Institute for Clinical Excellence (NICE) guidance in 2002, all designated breast cancer MDTs were reviewed by a team of clinical peers, and a total of 174 breast cancer MDTs were inspected as part of this 2004–2007 peer review round (National Institute for Clinical Excellence, 2002, 2008). There are currently no guidelines for acceptable sensitivity and specificity for diagnostic accuracy in such clinics.

The aim of this study was to calculate the number of patients discharged from a symptomatic breast clinic who develop breast cancer within the subsequent 3 years (effectively an interval cancer rate for the symptomatic service) and to determine how many of these cancers had been ‘missed’ at initial assessment.

## Materials and methods

All new patients referred to a specialist breast unit by their general practitioners from 1 January 2001 to 31 December 2003 were identified using a dedicated database. The investigation and management of patients were carried out according to local department protocols that have been adapted from national guidelines (Royal College of Radiologists, 2003; The Association of Breast Surgery, 2005). All patients underwent a careful clinical breast examination, and the degree of clinical suspicion was recorded using a 5-point scale (E1 normal–E5 clinically malignant). All patients over the age of 35 years underwent bilateral mammography, and any patient with a focal clinical or radiological abnormality has a targeted US. The imaging results were also recorded on a 5-point scale as for clinical examination. Any patient with clinically suspicious or focal solid abnormality underwent a core biopsy. All patients who underwent breast needle biopsy are subsequently discussed at a multidisciplinary meeting to decide on future management. All data are entered onto a dedicated breast database, the Joint Clinical Information System (JCIS), which is an *n*-tier web-based clinical information system supported by a SQL Server database. The system was built in-house in partnership with the breast clinical team using i5 Web application and Microsoft technologies including Visual Basic (Dataline Software Ltd, Brighton, UK). The system is designed to assist in the management and care of cancer patients by providing tools for clinicians to enter coded data at the point of care and to re-use that information for clinical notes, letters, waiting times performance management, clinical audit and research.

Each patient discharged during this period with a noncancer diagnosis was traced through the database for a further 3 years. Those patients who were subsequently diagnosed with breast cancer whether in the symptomatic clinic or breast screening programme were then identified. The age of the patient and length of time between initial presentation and subsequent cancer diagnosis were also recorded. The sensitivity of initial assessment examination was calculated as the proportion of cancers that were detected at the first assessment compared with the total number of cancers developing in each age group over the 3-year period. Correlation was also determined using histological findings of subsequent malignancy. The clinical details and imaging of the initial clinic visit were reviewed blinded to the clinical and imaging features of the subsequently diagnosed breast cancer. The clinician performed the breast examination, and the degree of clinical suspicion was noted. Mammographic breast density (according to the Breast Imaging Reporting and Data System (BI-RADS)) (D'Orsi *et al*, 2003), initial mammography and US report and any interventional procedures were noted. Imaging review was classified into one of the four categories according to the NHSBSP guidelines as follows: if imaging was unavailable, then imaging assessment was designated *unclassifiable*; normal/benign or *true interval* cancer if there were no suspicious features seen on prior imaging; uncertain or *minimal signs interval* cancer if mammographic changes were neither clearly benign or malignant; and suspicious or *missed* if the mammographic features were suspicious of malignancy (NHS Breast Screening Programme, 2005b). Thereafter, review was unblinded to assess whether uncertain or suspicious features were in the same breast and location as the subsequent diagnosis of cancer.

Following multidisciplinary review of both clinical and imaging investigations, a final symptomatic interval cancer classification was ascribed. These were as follows: *no delay* for patients presenting with a different problem and where review of previous clinical and imaging showed no suspicious findings; *probably no delay* for patients presenting with a different problem, but no images were available for review; *possible delay* for patients presenting with a different problem, but review of case showed subtle evidence of malignancy; *delay* in diagnosis (or ‘missed’) for patients presenting with the same problem that was subsequently diagnosed as breast cancer (whether imaging was available for review or not). For those patients whose imaging was unavailable for review, a designation of *probably no delay* or *delay* was given based on the available clinical information and imaging reports. The authors tended to err on the side of ‘worse’ classification (i.e., delay rather than probably no delay) so that the ‘miss’ rate was not underestimated.

Poisson regression was used to analyse categorical data, yielding likelihood-ratio *χ*^2^ tests, including tests for trend where data were ordered. Linear regression was used to analyse continuous end points, such as tumour size.

## Results

A total of 7613 new symptomatic patients were referred to the breast unit between 1 January 2001 and 31 December 2003, and of these, 609 patients (8.0%) were diagnosed with breast cancer. In total, 7004 patients were discharged from the clinic with a noncancer diagnosis, of whom, only 126 (1.8%) had further clinic appointments for a formal follow-up of their initial complaint. The remainder were discharged without follow-up; however, 2195 (31%) were re-referred by their general practitioner for further appointment(s) (ranging between 1 and 5 appointments). Of these patients, 29 were diagnosed with breast cancer in the 3 years after discharge and constituted the study cohort in this paper. All of these patients were diagnosed in the symptomatic clinic and none by screening mammography.

**Figure 1:**
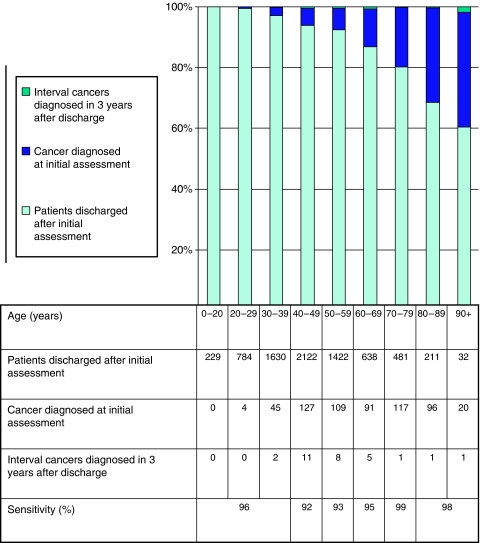
Graph showing the age distribution of 7004 patients discharged from a symptomatic breast clinic after an initial assessment, 609 patients diagnosed with breast cancer at the initial assessment and 29 patients diagnosed with an ‘interval’ cancer in the 3 years after discharge after an initial assessment. The sensitivity of initial assessment examination is derived from the proportion of cancers that were detected at the first assessment compared with the total number of cancers developing in each age group over the 3-year period.

The age distribution of each of these three groups is shown in Figure 1. Patients with breast cancer diagnosed at initial clinic visit and in the subsequent 3 years were significantly older than those discharged and remaining free of disease (*P*<0.001 and *P*=0.02, respectively). Patients with subsequently diagnosed cancer were younger (mean age 55 years) than those diagnosed at initial visit (mean age 62 years), although this did not reach statistical significance (*P*=0.07). Apart from the youngest ages, where numbers are extremely small, the sensitivity of the initial assessment for the diagnosis of cancer rises with age (see Figure 1). The lowest sensitivity of initial assessment was seen in women aged 40–49 years. Of the 29 patients who were subsequently diagnosed with breast cancer within 36 months, 6 were diagnosed within 12 months and 18 within 24 months. This equates to a symptomatic ‘interval’ cancer rate of 0.9 per 1000 women within 12 months, 2.6 per 1000 women within 24 months and 4.1 per 1000 women in the 36 months after the initial assessment (see Table 1). Time from initial assessment was not significantly related to patient tumour size, node status, grade, Nottingham Prognostic Index, breast density or oestrogen receptor status. There was a suggestive but nonsignificant finding that a longer interval was associated with greater age (*P*=0.09).

All available imaging performed at the time of initial assessment was reviewed by a panel of experienced breast radiologists, who were aware that the patient had developed breast cancer but not of its side or site. Imaging was unavailable for review in 14 (48%) patients. There were no suspicious features seen on prior imaging at the site of subsequent cancer development in 10 (35%) patients. There were minimal signs of malignancy in 4 (14%) patients (2 subtle parenchymal deformity, 1 small cluster of microcalcification and 1 mammographic asymmetry). The mammogram of one patient showed a small mass lesion away from the presenting clinical complaint that was overlooked at the initial assessment and was designated a suspicious or a ‘missed’ interval cancer.

**Figure 2:**
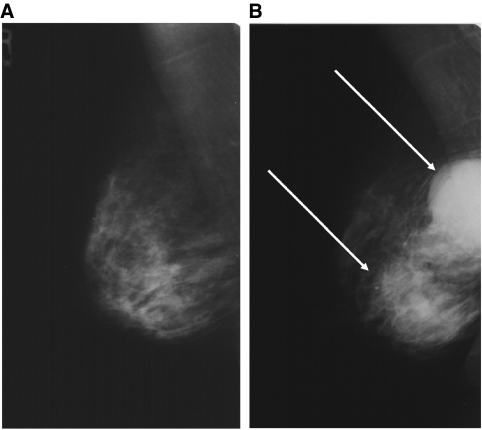
(**A**) The mammogram of a 58-year old patient referred with an 8-week history of a clinically benign (E2) lump inferior to the right nipple, reported as normal at initial assessment and subsequent review. A breast ultrasound (US) of the palpable lump by a consultant radiologist found no suspicious abnormality and the patient was discharged. (**B**) Mammogram taken 2 years later when the patient re-presented stating that the lump had enlarged and clinical examination showed a clinically malignant (E5) abnormality, shows an obvious large bilobed mass (arrows) suspicious for malignancy, which was confirmed on US. A core biopsy showed a metaplastic carcinoma.

The multidisciplinary final case classification was as follows: *no delay* in diagnosis in 10 (35%) patients; *probably no delay* in 7 (24%) patients; *possible delay* in 3 (10%) patients; and *delay in diagnosis* in 9 (31%) patients (Table 2 and Figure 2). The likelihood of delay in diagnosis was not significantly associated with patient's age or breast density. Tumours where a delay in diagnosis was suspected were significantly larger (mean 33 *vs* 23 mm diameter; *P*=0.03) and had significantly more involved nodes (median of 1 *vs* 6 nodes; *P*=0.03), compared with those where no delay was suspected. Grade and oestrogen receptor status were not significantly different.

The initial presentations of the 12 patients with *delay* or *possible delay* in diagnosis were varied (lump 9, nipple discharge 1, breast pain 1 and nipple eczema 1). Clinical examination was performed by a consultant nurse practitioner or consultant breast surgeon in 8 (67%), surgical trainee in 3 (25%) and an experienced associate specialist in breast disease in 1 (8%) patient. Clinical examination findings in the breast subsequently diagnosed with cancer were designated normal (E1) in 2, benign (E2) in 8 and suspicious probably benign (E3) in 2 patients. None had more suspicious findings. All patients underwent bilateral mammography as part of their initial imaging assessment. The breast density was BI-RADS category 1 (<25% fibroglandular tissue) in 1, category 2 (26–50% fibroglandular tissue) in 1, category 3 (51–75% fibroglandular tissue) in 6 (55%) and category 4 (>75% fibroglandular tissue) in 4 patients. Mammographic interpretation of the breast that subsequently was diagnosed with breast cancer was reported as normal in 8 (67%) and benign in 4 (33%) patients. Ultrasound was performed in eight patients, of which four were reported normal and four were benign. The radiological examination was undertaken and reported by a consultant breast radiologist in 10 (83%) and by a supervised radiologist in training in 2 (17%) patients. One patient underwent a punch biopsy of the nipple and one a cytological smear of nipple discharge all of which produced benign results.

## Discussion

We have calculated the interval cancer rate for patients discharged from a symptomatic breast clinic with a nonmalignant diagnosis. The ability to carry out this audit, and the high quality of the data, has only been possible as a result of prospective data collection using the JCIS, an electronic record for cancer patients. Although the population is different from that seen in a screening programme (largely women between 50 and 70 years in the NHSBSP), it is of interest to compare the relative interval cancer rates. The expected standard target in the NHSBSP for interval cancer rate is <1.2 per 1000 women screened in the first 2 years and 1.4 per 1000 women screened in the third year (NHS Breast Screening Programme, 2005b). Reported interval cancer data from Breast Test Wales is 0.7 per 1000 women screened in the first year, 1.3 in the second and 1.6 in the third year (Breast Test Wales, 2005). The equivalent rates from our symptomatic population are very similar to these data at 0.9 per 1000 women in the first year, 1.7 within the second year and 1.6 in the third year after an initial assessment (see Table 1). As these data are from a single unit, any patient developing breast cancer after discharge and presenting to another centre would not be included resulting in an underestimate of the interval cancer rate. As the local population demographic is one of net influx, this should mitigate against this being a significant under ascertainment. These figures equate to a diagnostic accuracy of 99.6%. It is important to note that all patients undergoing needle biopsy were discussed at a multidisciplinary meeting in contrast to many breast units where only breast cancers are discussed. This allows time for discussion of difficult diagnostic cases and almost certainly contributes to this very high level of diagnostic accuracy. The choice of biopsy technique may well also be important. During this study period, wide bore needle core biopsy with histopathological diagnosis was the only method used having been shown earlier to be superior to fine needle aspiration cytology (Britton, 1999).

Were mistakes made at initial assessment that might have contributed to a delay in diagnosis? Over 80% of the patients who developed an interval cancer were examined by experienced consultants or specialist practitioners. Although this reflects our practice of the clinical examination not being performed by trainees without very close supervision, our policy has become more rigorous since that described in the initial study period of 2001–2003. Any individual now undertaking clinical breast examination has to carry out an audit of breast and axillary examination of at least 500 patients and requires a 90% concordance with a consultant examination (Chapman *et al*, 2002). This section of the advanced practitioner course often forms part of an MSc degree. Similarly, a consultant breast radiologist supervised the imaging in all 29 patients and directly performed the examination in over 80% of the patients. Only 1 (3%) patient had a definite abnormality that was overlooked on initial mammographic examination and the images of 4 (14%) patients showed subtle abnormalities. It is unfortunate, however, that imaging for 14 (48%) of the patients was unavailable for review and reflects a policy of aggressive film culling within the NHS because of lack of the film storage space. As stated earlier, the authors tended to err on the side of ‘worse’ classification (i.e., delay rather than probably no delay) so that the ‘miss’ rate was not falsely underestimated. However, the missing images do weaken the robustness of the final case classification. The authors felt that the lack of images should not exclude a patient from being included in the series, as this would result in a misleadingly underestimate of the missed cancer rate.

We have calculated the sensitivity of the initial assessment as the proportion of cancers that were detected at the first assessment compared with the total number of cancers developing in each age group over the 3-year period. These data will include cancers that were either not present or undetectable at the time of initial assessment. The lowest sensitivity of initial assessment was seen in women aged 40–49 years. This is not surprising as patients in this age group, with denser breasts and higher levels of benign pathology, present the greatest imaging and diagnostic challenges. This is well illustrated by the fact that 90% of the interval cancer patients had dense or very dense mammograms.

A multidisciplinary meeting reached a consensus decision that just over 30% of patients developing an interval cancer after a symptomatic assessment did present with the same problem as was subsequently diagnosed as breast cancer resulting in a delay in diagnosis. An additional 10% of patients had a possible delay in diagnosis.

Unlike many breast units, all patients in this series were seen in a one-stop multidisciplinary clinic with access to same day imaging and biopsy. A key part of the Cancer Reform Strategy (UK Department of Health, 2007) is a maximum 2-week wait for all breast referrals by December 2009, and there is no doubt that the provision of one-stop assessment for all patients can facilitate this. The data from this audit can reassure both clinicians and patients that one-stop breast clinics provide high-quality diagnostic accuracy, with very few missed cancers, and contribute to early breast cancer diagnosis and treatment.

## Summary

We have shown that triple assessment carried out in an MDT setting is extremely safe with an overall diagnostic accuracy of 99.6%. The lowest sensitivity for initial assessment occurs in younger women aged 40–49 years. For every 1000 patients seen and discharged, just over 4 will return and be diagnosed with breast cancer in the following 3 years. Of these, 1.3 will have been unequivocally missed at the initial presentation and a further 0.4 will have had a subtle imaging abnormality that was overlooked at the initial assessment. The ‘missed’ cancer rate overall is therefore 1.7 per 1000 women discharged over a 3-year period.

## Figures and Tables

**Table 1:** The histopathological findings and length of time from initial assessment of 29 patients who were diagnosed with breast cancer having been previously discharged from the breast clinic.

				**Histopathology of interval cancer**					
**Interval between initial assessment and interval cancer (months)**	**No. of patients**	**Mean age years (range)**	**Mammographic density (BI-RADS 1–4)**	**Mean size mm (range)**	**Grade**	**Nodal status^a^**	**ER status**	**% of patients with NPI >3.4 (moderate/poor/very poor prognostic groups)^b^**	**Interval cancer rate per 1000 patients discharged from a symptomatic clinic**
0–12^c^	6	49 (39–56)	1: 0 2: 0 3: 3 4: 3	29 (8–52)	Gd 1: 0 Gd 2: 2 Gd 3: 3	1: 2 2: 1 3: 2	+ve 4 −ve 1	60	0.9
13–24	12	57 (41–96)	1: 2 2: 1 3: 3 4: 6	29 (9–60)	Gd 1: 1 Gd 2: 4 Gd 3: 7	1: 4 2: 4 3: 4	+ve 6 −ve 6	91	1.7
25–36	11	56 (38–87)	1: 0 2: 0 3: 10 4: 1	25 (18–40)	Gd 1: 1 Gd 2: 2 Gd 3: 8	1: 5 2: 4 3: 2	+ve 8 −ve 3	91	1.6
Total^c^	29	55 (38–96)	1: 2 2: 1 3: 16 4: 10	27 (8–60)	Gd 1: 2 Gd 2: 8 Gd 3: 18	1: 11 2: 9 3: 8	+ve 18 −ve 10	86	4.1

a According to the Nottingham Prognostic Index (Blamey *et al*, 2007a, 2007b).

b NPI indicates Nottingham Prognostic Index (Blamey *et al*, 2007a, 2007b).

c Final histology unknown in one patient who emigrated and underwent treatment abroad.

**Table 2:** The histopathological findings and classification of whether a delay in diagnosis had occurred of 29 patients who were diagnosed with breast cancer having been previously discharged from the breast clinic.

						**Histopathology of ‘interval’ cancer**					
**Classification of interval cancer**	**No. of patients**	**Mean age years (range)**	**Mean time from initial discharge to diagnosis of breast cancer (months)**	**Mammographic density (BI-RADS 1–4)**	**Imaging review classification^a^**	**Mean size mm (range)**	**Grade**	**Nodal status^b^**	**ER status**	**% of patients with NPI >3.4 (moderate/ poor/very poor prognostic groups)^c^**	**Interval cancer rate per 1000 patients seen in a symptomatic clinic**
No delay	10	50 (41–56)	19.4	1: 0 2: 0 3: 6 4: 4	10 Normal/benign	20 (8–33)	Gd 1: 1 Gd 2: 3 Gd 3: 6	1: 5 2: 3 3: 2	+ve 5 −ve 5	70	1.4
Probably no delay	7	58 (39–87)	23.6	1: 1 2: 0 3: 4 4: 2	7 Unclassifiable	29 (17–50)	Gd 1: 0 Gd 2: 0 Gd 3: 7	1: 4 2: 3 3: 0	+ve 5 −ve 2	100	1.0
Possible delay	3	59 (53–69)	17.3	1: 0 2: 1 3: 1 4: 1	3 Uncertain	33 (16–52)	Gd 1: 1 Gd 2: 1 Gd 3: 1	1: 1 2: 1 3: 1	+ve 3 −ve 0	66	0.4
Delay^d^	9	57 (36–96)	21.8	1: 1 2: 0 3: 5 4: 3	7 Unclassifiable 1 Uncertain 1 Suspicious	33 (20–60)	Gd 1: 0 Gd 2: 4 Gd 3: 4	1: 1 2: 3 3: 4	+ve 5 −ve 3	100	1.3
Total^d^	29	55 (38–96)	20.9	1: 2 2: 1 3: 16 4: 10		27 (8–60)	Gd 1: 2 Gd 2: 8 Gd 3: 18	1: 11 2: 9 3: 8	+ve 18 −ve 10	86	4.1

a Imaging Review Classification: Unclassifiable (imaging unavailable for review), normal/benign (no suspicious features), uncertain (neither clearly benign or malignant imaging changes), suspicious (‘missed’ cancer).

b According to the Nottingham Prognostic Index (Blamey *et al*, 2007a, 2007b).

c NPI indicates Nottingham Prognostic Index (Blamey *et al*, 2007a, 2007b).

d Final histology unknown in one patient who emigrated and underwent treatment abroad.
